# Pan-Resistome Insights into the Multidrug Resistance of *Acinetobacter baumannii*

**DOI:** 10.3390/antibiotics10050596

**Published:** 2021-05-18

**Authors:** Diego Lucas Neres Rodrigues, Francielly Morais-Rodrigues, Raquel Hurtado, Roselane Gonçalves dos Santos, Daniela Camargos Costa, Debmalya Barh, Preetam Ghosh, Khalid J. Alzahrani, Siomar Castro Soares, Rommel Ramos, Aristóteles Góes-Neto, Vasco Azevedo, Flávia Figueira Aburjaile

**Affiliations:** 1 Laboratory of Cellular and Molecular Genetics, Universidade Federal de Minas GeraisBelo Horizonte, Belo Horizonte 31270-901, MG, Brazil; dlnrodrigues@ufmg.br (D.L.N.R.); franrodriguesdacosta@ufmg.br (F.M.-R.); raquelgen1@gmail.com (R.H.); roselanegr@gmail.com (R.G.d.S.); dr.barh@gmail.com (D.B.); arigoesneto@icb.ufmg.br (A.G.-N.); faburjaile@gmail.com (F.F.A.); 2 FAMINAS-BH, Belo Horizonte 31744-007, MG, Brazil; daniela.costa@faminasbh.edu.br; 3 Institute of Integrative Omics and Applied Biotechnology, Nonakuri West Bengal 721172, India; 4 Department of Computer Science, Virginia Commonwealth University, Richmond, VA 23284, USA; preetam.ghosh@gmail.com; 5 Department of Clinical Laboratories Sciences, College of Applied Medical Sciences, Taif University, P.O. Box 11099, Taif 21944, Saudi Arabia; ak.jamaan@tu.edu.sa; 6 Department of Research Development and Technological Innovation, Universidade Federal do Triângulo Mineiro, Uberaba 38025-180, MG, Brazil; siomars@gmail.com; 7 Faculty of Biotechnology, Universidade Federal de Pará, Belém 66075-110, PA, Brazil; rommelramos@ufpa.br

**Keywords:** antimicrobial, drug resistance, pan-genome, multilocus sequence typing, nosocomial infections

## Abstract

*Acinetobacter baumannii* is an important Gram-negative opportunistic pathogen that is responsible for many nosocomial infections. This etiologic agent has acquired, over the years, multiple mechanisms of resistance to a wide range of antimicrobials and the ability to survive in different environments. In this context, our study aims to elucidate the resistome from the *A. baumannii* strains based on phylogenetic, phylogenomic, and comparative genomics analyses. In silico analysis of the complete genomes of *A. baumannii* strains was carried out to identify genes involved in the resistance mechanisms and the phylogenetic relationships and grouping of the strains based on the sequence type. The presence of genomic islands containing most of the resistance gene repertoire indicated high genomic plasticity, which probably enabled the acquisition of resistance genes and the formation of a robust resistome. *A. baumannii* displayed an open pan-genome and revealed a still constant genetic permutation among their strains. Furthermore, the resistance genes suggest a specific profile within the species throughout its evolutionary history. Moreover, the current study performed screening and characterization of the main genes present in the resistome, which can be used in applied research to develop new therapeutic methods to control this important bacterial pathogen.

## 1. Introduction

*Acinetobacter baumannii* is a Gram-negative bacterium, aerobic, non-fermenting, catalase-positive coccobacillus with cosmopolitan distribution [1,2]. Most of the clinical cases involving this bacterial species are related to one or more of the following pathological conditions: severe pneumonia, meningitis, bacteremia, and erysipelas [1,3,4,5]. Although members of the genus *Acinetobacter* are ubiquitous, they are rarely isolated in the environment outside of hospitals, even during outbreaks [6].

This species has several intrinsic resistance mechanisms, such as (I) the presence of β-lactamases, which is responsible for the degradation of β-lactam drugs; (II) the presence of multiple drug efflux pumps that prevent the increase in the concentration of antimicrobials in the cytoplasm; (III) changes in the molecular pattern of proteins associated with plasma membrane; (IV) ribosomal methylation, which hinders the action of antimicrobials related to the regulation of protein translation processes, such as tigecyclines and quinolones; and (V) the presence of enzymes capable of degrading multiple antimicrobials [7,8,9].

The recommended treatment usually prescribed for infections with *A. baumannii* is based on β-lactam antibiotics, such as cephalosporins and carbapenems [10]. This class of antibiotics interferes with peptidoglycan biosynthesis and avoids forming the cell wall [11,12]. Nonetheless, over time, because of its high adaptation skills, strains capable of resisting the high concentrations of these antimicrobials have been detected [2,8]. Such cases of resistance can be classified into three categories: (i) Extensively Drug Resistant (XDR) refers to when it is resistant to more than three classes of antimicrobials; (ii) Multidrug Resistant (MDR), when it is resistant to almost all the antimicrobials except for two; or (iii) Pandrug Resistant (PDR), when it is resistant to all known antimicrobials.

Because of all these factors associated with the ability of *A. baumannii* to survive to adverse conditions (grow under a wide thermal range and in an environment with low concentrations of nutrients) and the resistance exhibited by *A. baumannii* generates numerous obstacles for the hospital treatment team, making it difficult to treat patients [1,4,6,9,13]. Some resistance mechanisms of protein origin have been previously evidenced, such as changes in the DNA-gyrase complex and an increase in the expression of the *ampC* that confers carbapenem resistance to *A. baumannii* [8].

In order to better clarify some of the resistance mechanisms present in the species genome, this study explores the genes occurring on the resistome of 206 complete genomes of strains of *A. baumannii* that are related to the resistance of this species by using multi-omic methodologies for comparative genomics, phylogenomics, and the pan-resistome of this species.

## 2. Results

### 2.1. Genomic Analysis and Geographic Distribution

*Acinetobacter baumannii* is a genetically diverse bacterial species and there is a variety of typing methods to identify genetic differences among the strains that could be associated with pathogenicity, epidemiological origin, dissemination, and evolutionary patterns [14]. Sequence type and phylogenetic analysis allow for the identification of genotype groups with a phylogenetic relationship and explore the diversity among the strains [15]. Similarity nucleotides and MLST analysis with geographical data can reveal a better knowledge of the epidemiological context and population structure among the strains around the world [16,17]. With the analysis of genomic similarity based on sequence alignment and geographic distribution, it is possible to infer bacterial clonality, considering that strains of bacterial species isolated from the same region tend to have the same genic repertoire. Even though events of gene drift and vertical gene transfer cannot be ruled out, genetic characteristics are generally conserved when dealing with isolated bacteria in the same site or nearby sites.

Numerous epidemiological studies of *A. baumannii* associate it with the presence of ST by local origin, as seen in the occurrence of ST 848 (CC 208) (Oxford scheme) carrying resistance gene to carbapenems in India [18], and likewise the frequent presence of ST15, ST25, ST79, and ST1 in South America [19,20]. A recent phylogeographical analysis of the Italian isolates belongs to the only clonal group ST78 (Pasteur scheme) [14].

The 206 *A. baumannii* complete NCBI genomes sequences were analyzed (see Supplementary Table S1). The genomes have sizes varying from 3.48 Mb to 4.43 Mb, with a genomic GC content of 39.05%. Considering that nearby isolated bacterial genomes tend to maintain the same genetic characteristics, the study of the geographical distribution of *A. baumannii* is an essential method for evaluating the conservation of the species in the global context.

**Figure 1:**
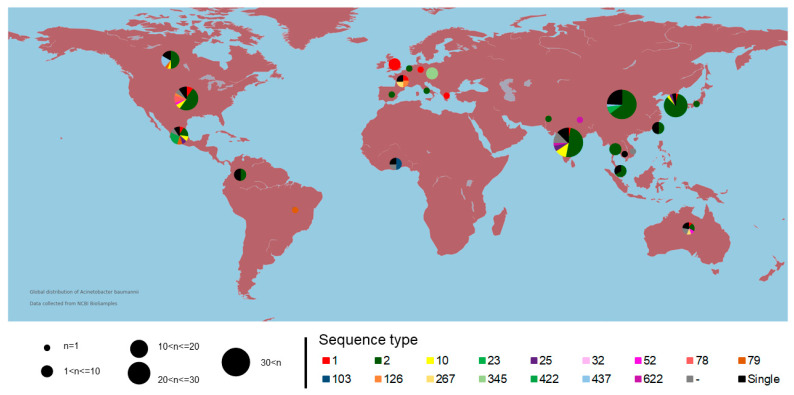
Graphical representation of the global distribution of isolation sites of different strains of *Acinetobacter baumannii* in a grouped way. The colors represent the sequence types of the strains in this study. The size of the circle indicates the number of isolated strains.

It is important to note that all strains added to the study showed similarities greater than 95% based on the ANI results (see Supplementary Figure S1). This result corroborates the statement that all strains belong to the same species [21,22]. A total of five relevant clusters with high similarity (≳98.5%) belonging mainly to specific STs (1, 2, 10, 79, and 437) were retrieved. This finding corroborates the conservation of genomes belonging to the same ST. Consequently, strains related to the same ST were expected to be isolated at locations to justify the high genomic similarity. Nevertheless, the geographic distribution of the strains according to the ST proved to be misplaced. Considering that different STs were isolated on distinct continents, possible factors that could justify this misplacing are microbial ubiquity and globalization (Figure 1). A higher number of deposited genomes belong to ST 2 (50% of the used dataset) and a more significant number of strains were isolated from the Asian continent (51.2% of the used dataset). These data do not corroborate the epidemiological information on the distribution of outbreaks caused by the bacterium *A. baumannii* [9,15,18]. Thus, this concludes that there is a more significant number of sequencing performed on the Asian and North American continents since epidemiological outbreaks have been reported in several developing countries over time (Argentina, Brazil, and South Africa). Furthermore, this pathogen has also reported outbreaks of infections on the European continent; however, the number of isolates from that continent is still much lower.

### 2.2. Phylogeny and Phylogenomics

Phylogenetically, all the *A. baumannii* strains were grouped in the same clade within the *Acinetobacter* genus, confirming the monophyly of this species (see Supplementary Figure S2). This result also points out that the *A. baumannii* strains are highly conserved within the species. It is also observed in different microbial species and is consistent with reports from the literature on phylogenetic analysis, indicating that the use of housekeeping genes to infer evolutionary history is a good qualifier of phylogenetic distance and epidemiology [23].

Three strains (FDAARGOS_494, FDAARGOS_493, and FDAARGOS_560), previously identified as *Acinetobacter* sp., were grouped together and inside the *A. baumannii* clade, strongly suggesting that they are, in fact, of this same species. This taxonomic re-classification has already occurred in other cases of bacterial species [24,25,26]. More phylogenomic studies, including tetranucleotide analyses, Average Nucleotide Identity (ANI), and the presence and absence of species-specific genes evaluation, are needed to confirm this hypothesis and assure taxonomic reclassification based on genomic data and theoretical background [24,27]. These three strains were not added to the subsequent analyses. The genomic similarity analysis integrated with a previous phylogenetic analysis was ideal for determining the exclusive addition of *A. baumannii* strains to the following in silico analysis, ensuring that the pan-genomic analyses were not skewed.

**Figure 2:**
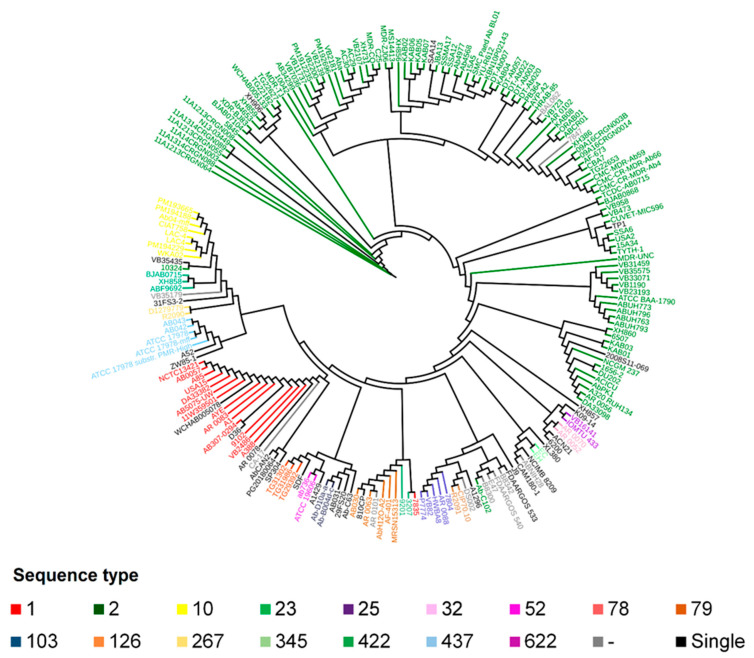
Phylogenomic tree based on the core genome of 206 *Acinetobacter baumannii* strains. The colors represent the grouping by sequence type. The method used was maximum likelihood with statistical support of 1000 bootstraps with 1999 genes present in the core genome.

The *A. baumannii* strains were grouped according to their respective STs in the phylogenomic tree, using the core genome sequence (Figure 2). Nonetheless, in the phylogenomic analyses, the ST 2 strains (represented in green) formed paraphyletic clades, and, thus, these strains cannot be considered to be in the same group. The strains represented in gray do not have a defined ST, but they all grouped in the same clade, indicating the high similarity among them (see Supplementary Figure S1).

### 2.3. Genomic Plasticity

**Figure 3:**
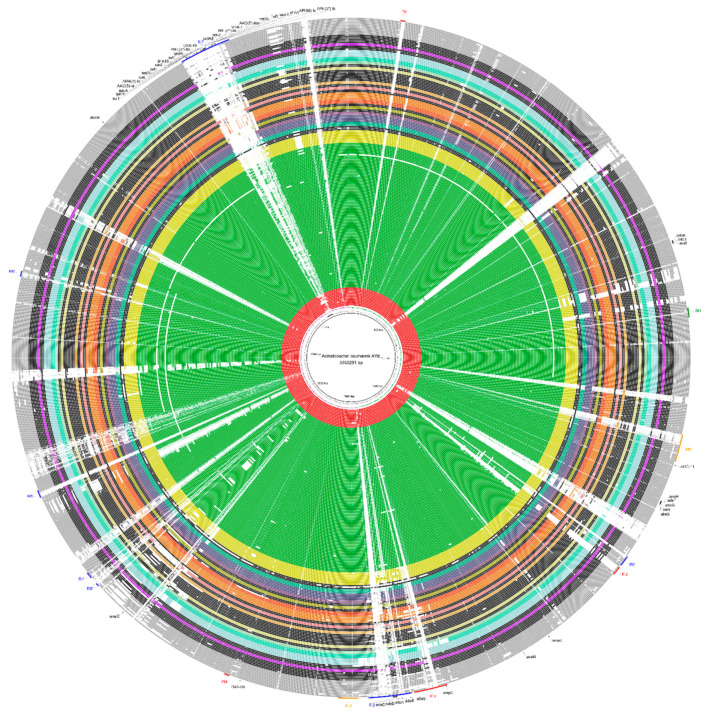
Representation of the circular genome of the *A. baumannii* AYE strain as a central genome. The compared strains were grouped and colored.

During the analysis of genomic plasticity, a significant gap in the *A. baumannii* strains could be observed when visually compared. Even strains belonging to the same ST were not identical, although they were genomic, phylogenomically closer, and shared the same clade. This result suggests that the strains of this species are not very clonal and tend to have a high rate of gene permutation since there are many gaps between the genomes (Figure 3).

Comparative genomic analyses of the 206 *A. baumannii* genomes, using the AYE strain as a reference, showed the presence of 14 genomic islands (Figure 3). Among these 14 genomic islands, 4 were Pathogenicity islands, 2 were Metabolic islands, 1 was a Symbiotic island, and 7 were Resistance islands. Furthermore, 1 full-sized Resistance island (RI7 or AbaR1) was identified within the AYE strain. This genomic region has a length of 96,878 nucleotides and contains the highest amount of resistance genes found in this species. There are 25 resistance genes within this island divided into efflux pumps and proteins with enzymatic activity.

The islands RI2 (80,220 bp) and RI7 (96,878 bp) were conserved within the species, which were more present within strains belonging to ST 1. Outside of this cluster, however, both islands were not entirely found. A similar result was observed in smaller islands, such as RI1 (20,317 bp), RI3 (6077 bp), RI4 (12,534 bp), RI5 (14,763 bp), and RI6 (10,374 bp), indicating that they are unstable regions within the genome.

There is a great number of genomic islands for the *A. baumannii* species, which reveals its high genomic plasticity. Although we identified a reduced number of type sequences and phylogenetically close strains, analyzing the complete genomes showed how all the strains are different in their gene content. This could be due to the horizontal acquisition of mobile genetic elements or gene duplication events.

### 2.4. Analysis of the Pan-Genome for Understanding This Species

There is an intensive effort to know the total repertoire of the *A. baumannii* species. Classically, the pan-genome assesses the total gene repertoire of a sample, population, or species. To this end, it considers subpartitions of the complete set, which are (I) a core genome consisting of genes shared by all the strains analyzed; (II) an accessory genome consisting of genes shared by two or more strains analyzed, but not all strains; (III) singletons (or exclusive genes), characterized to present exclusively in a single strain [28].

As a result, according to the Heaps’ law, the pan-genome of *A. baumannii* remains open (α = 0.71), which by each newly added genome, the number of new genes will increase the genetic repertoire of the species. This result was obtained using the formula $n=a\times x^{1-\alpha }$, where *n* is the estimated size of the pan-genome for a given number of genomes, *x* is the number of genomes used, and α is a fitting parameter [28]. As a rule, when 0 < α < 1, the pan-genome is considered open. This fact also corroborates the high genomic plasticity already reported for this species, especially considering that this bacterium has an exceptional ability to obtain new gene content through transposable elements [14,29].

**Figure 4:**
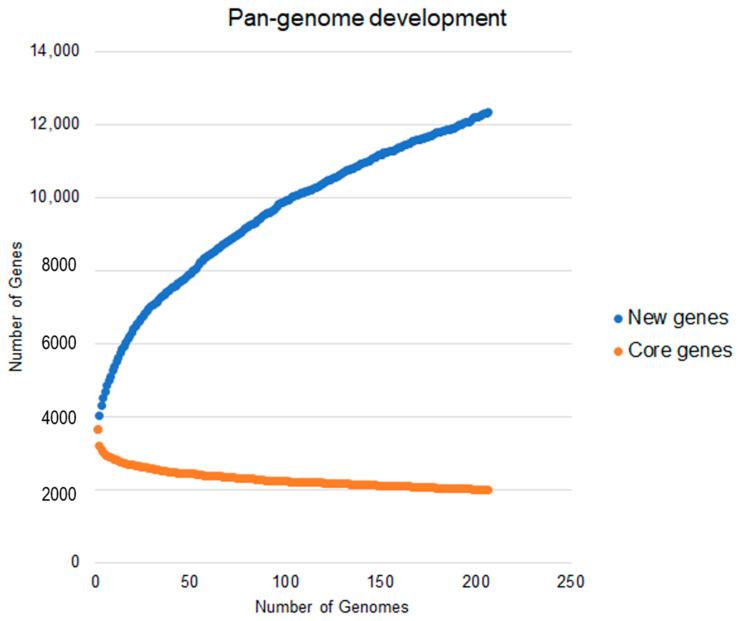
Development curve of the pan-genome of *Acinetobacter baumannii*.

The pan-genome analysis revealed a total of 12,336 genes, of which 1999 genes are shared for all strains (complete genome sequences of *A. baumannii*), and 3920 were strain-specific genes. The accessory genome, except for single genes, is made up of 6417 genes. Figure 4 represents the development of the *A. baumannii* pan-genome. It is possible to observe that even using 206 genomes, the curve did not reach a point of stability or a plateau. This fact corroborates the alpha value found, as it also indicates an open pan-genome.

The different patterns of the presence of genes of the SDF strain can be observed in a detailed analysis. This strain is already known to be susceptible to antimicrobials and is the only representative of sequence type 17. Its accessory gene pattern differs from all the others and has about 362 unique genes, which contrasts with the pattern of the super-resistant AYE strain, which contains about 11 unique genes. This fact, combined with the distant phylogenomic position of the strain, shows how different the susceptible strain is from the others.

**Figure 5:**
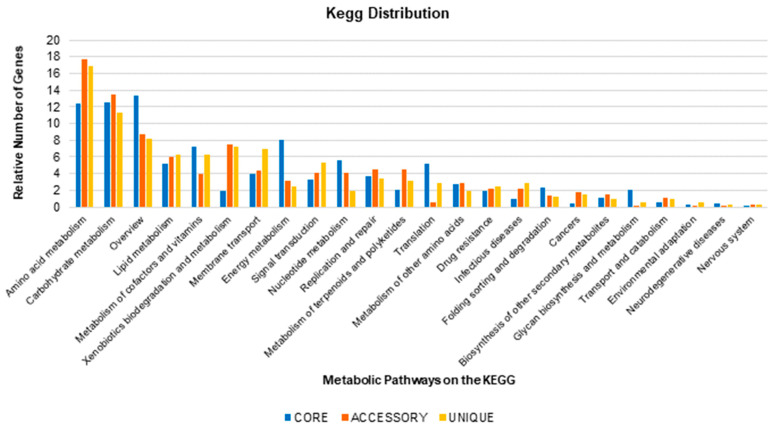
Graphical representation of the gene distribution by metabolic pathway within each subpartition of the total pan-genome. Only pathways with at least 0.1% of the genes represented in each subpartition of the pan-genome were considered.

A more accurate analysis of the total pan-genome indicates the number of genes related to specific bacterial metabolic pathways. Such analysis is based on the KEGG database. It demonstrates a high number of core genes related to metabolic pathways intrinsic to microbial existence, such as energy metabolism (8.00%) and molecular translation (5.16%) (Figure 5). The accessory genes are related to amino acid metabolism (17.64%), carbohydrate metabolism (13.42%), and xenobiotics biodegradation and metabolism (7.51%). Most of the genes related to drug resistance are part of the accessory genome (2.24%), compared to their percentage represented in the core genome (1.89%). Similarly, genes related to infectious diseases are represented in the core genome (0.94%), accessory genome (2.24%), and strain-specific genes (2.82%).

As for genes related to adaptation to the environment, there is a very low gene repertoire associated with this process in the general pan-genome, with less than 1.0% of the total repertoire linked to such a pathway in any subdivision of the pan-genome.

### 2.5. Pan-Resistome Characterization of Acinetobacter Baumannii

A pan-resistome analysis contains analogous divisions applied to a pan-genomic analysis, but focused on microbial resistance factors [30]. Considering a similarity criterion greater than 70% and an E-value < 5 × 10^−6^, all the studied strains present a pan-resistome of 171 genes, and within that, a core resistome constituted of 9 genes is shown in Table 1 [11].

In these analyses, the strains that presented *ade*-type bombs were expected to have the complete gene repertoire to be functional. Nevertheless, this pattern was observed exclusively for the *ade*IJK efflux pump, as all the genomes presented the genes *adeI*, *adeJ*, and *adeK*. However, the same pattern was not observed for the other genes of the same family (see Supplementary Figure S3 and Supplementary Table S2). Similarly, to the genes capable of constituting the *ade*FGH pump, the presence only of the *ade*F and *ade*G genes was detected in all the strains. The gene *ade*H (the outer membrane factor protein in the *ade*FGH multidrug efflux complex) was not found in three strains (XDR-BJ83, ORAB01, and DS002), and, in theory, makes the activity of the pump unfeasible. Our study also identified an interesting protein present in all strains: the *ampC* enzyme. This is responsible for generating resistance to beta-lactams, specifically cephalosporin, and is thought to cause hydrolysis of the drug [31,32].

Analyzing the accessory portion of the resistome, an interesting distribution profile of specific genes was retrieved. The OXA-66 gene, responsible for coding a variant of beta-lactamase with action against penam, carbapenem, and cephalosporin, for example, was present in 99 strains, which is equivalent to approximately 48% of the dataset. Among these, 93 belonged to the ST 2. This fact makes this gene almost exclusive to strains belonging to ST 2. Regarding the other ST, only six strains had the OXA-66 gene, and they do not belong to ST 2, which are BAL062—ST unknown; SAA14—ST 187; XH857—ST 215; XH906—ST 922; 7847—ST unknown; TP1—ST 570.

A similar pattern was observed with the ADC-76 gene, responsible for encoding a beta-lactamase that caused cephalosporin inactivation and that was present in strains belonging exclusively to STs 23, 10, 85, 464, 575, and 639. The same was true for the OXA-68 gene, identified only in strains belonging to STs 23 and 10 but not present in all the strains. The same went for the OXA-180 gene, which was detected only in strains of ST 267. The gene responsible for encoding OXA-69 was almost exclusive to strains belonging to STs 1, 20, 81, and 195.

Other different patterns of gene distribution can be seen in Supplementary Table S2. Nonetheless, there was no significant pattern of visible distribution related to the geographic location of the isolates, except in some cases. The OXA-67 gene was exclusive to isolates (strains EC and EH) from the Czech Republic, while the ADC-81 and OXA-92 genes were observed only in the A388strain.

Otherwise, the presence of plasmid content in *A. baumannii* is already known. Among the 206 strains selected for the study, 162 were deposited with the plasmid sequence. However, there was no statistical difference regarding the number of resistance genes in strains with plasmid versus strains that did not show plasmid (*p*-value = 0.3081). However, qualitative differences were expected. As an example, 21 genes were found exclusively in plasmids (see Supplementary Table S3). Among the 21 exclusive plasmid genes was the MCR-4.3 gene, the only one predicted in the entire pan-resistome with action against polymyxins.

**Figure 6:**
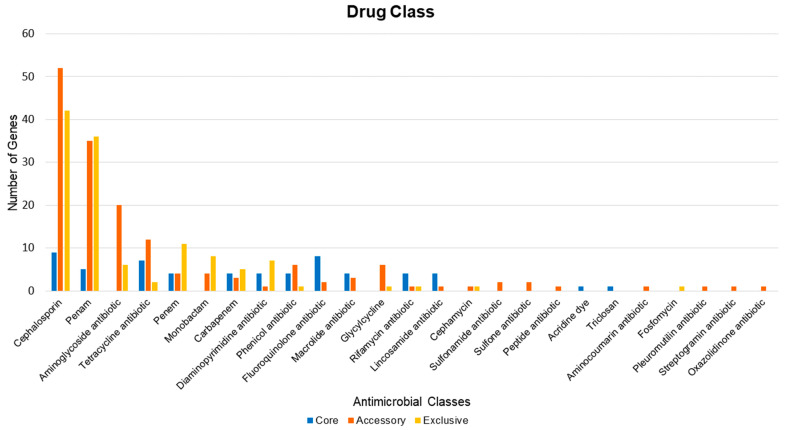
Distribution of the resistance mechanisms related to each antimicrobial found in the database used in the predicted total pan-resistome.

As the distribution related to the number of antibiotics was linked to each subpartition of the pan-resistome, the antibiotic with the highest amount of resistance mechanisms linked to it was cephalosporin, with about 103 resistance proteins within the formed pan-resistome (Figure 6). In contrast, antimicrobials (sulfonamide, sulfone, cephamycin, and pleuromutilin) had low amounts of resistance mechanisms related to the predicted resistome of *A. baumannii*.

**Figure 7:**
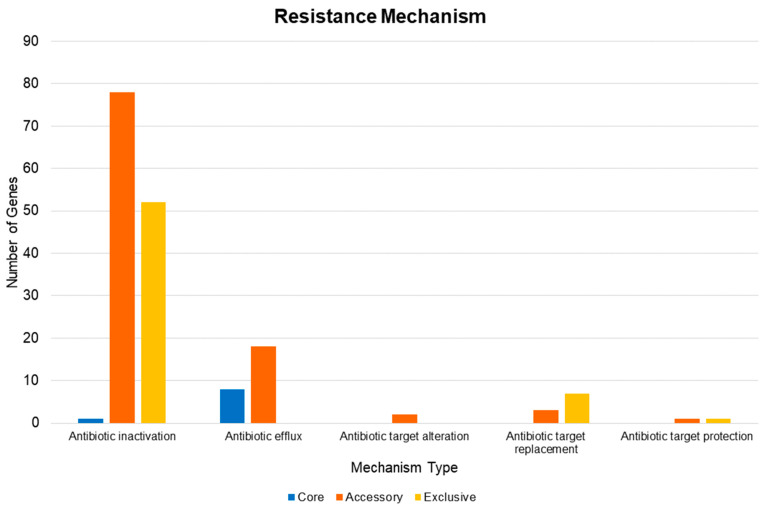
Distribution of the resistance mechanisms related to each type of action provided by the translated protein.

In accordance with the distribution of the types of resistance mechanisms found, 131 caused the enzymatic inactivation of the antibiotic (Figure 7). This total is equivalent to 76.6% of the predicted pan-resistome. Moreover, almost all the core resistome-related proteins are efflux pumps (8 proteins).

The genomics islands of resistance identified some genes, such as adeS, adeR, adeA, adeB, and adeC (within resistance island 2). Moreover, on resistance island 7 (or AbaR1 island), the following antimicrobial resistance-related genes and products were detected: sul1, qacH, AAC(3)-Ia, APH(3′)-Ia, catI, tet(A), dfrA10, ANT(3″)-IIa, OXA-10, cmlA5, arr-2, ANT(2″)-Ia, VEB-1, AAC(6′)-Ian, tet(G), floR, dfrA1, APH(6)-Id, and APH(3″)-Ib.

## 3. Discussion

### 3.1. Similarity Analysis, Geographic Distribution, and Phylogenomic Reconstruction

Recently, a more significant number of sequences of the *A. baumannii* genome provided resources for studying genomic epidemiology. Out of the 206 genomes analyzed, we identified 47 unique STs, of which STs 1, 2, 18, and 79 were distributed in significant prevalence throughout North America, Europe, and the East Asian continent, but also a diverse set of STs was indistinctly spread across the globe. The presence of few STs could be because most of the genome sequencing projects come from a single outbreak or several strains representing the same geographic location, and, in some cases, a unique ST was reported by geographic information.

According to Jeannot et al. (2014), there is a higher prevalence of strains belonging to ST 2 across the globe, but mainly in the European continent. The previous work also pointed out the polyclonality of *A. baumannii* strains within the French nation, considering the existence of different STs randomly isolated throughout the country [15].

Based on the analysis of sequence similarity of STs among the global distribution of *A. baumannii* strains, we reported several genogroups (STs) with genomic similarity less than a 98% identity in the same geographic region (Figure 1 and Supplementary Figure S1), which is opposite to the initial hypothesis that isolates from the same region exhibit high genomic similarity [23]. We also observed that the obtained result revealed a discrepancy for strains with the same ST (Figure 1 and Supplementary Figure S1). Strains from different STs in the same geographic region and the same STs isolated on other continents may maintain high similarity (>99%). In contrast, diverse genomic strains, with less than a 98% identity, are shared in nearby locations.

In previous studies, Kazmierczak and et al. (2016) reported a heterogeneous global distribution between strains of *Pseudomonas aeruginosa* and *Enterobacter* spp. based on genetic variants of lactamases. In our study, geographically close isolates may or may not have the same variant. Consequently, phylogenetic proximity is not mandatory for members of the same geographically close species [33].

The phylogenomic tree of 206 genomes displays concordance with the ST distribution, which is expected since both analyses depend on the vertical evolutionary relationship among strains. The tree phylogeny and distribution of STs, however, show a relationship with the geographical origin. This would indicate that the dissemination of *A. baumannii* does not present a population structure. Nevertheless, it is not definitive because a higher and representative number of strains is required to evaluate the population structure.

It is biologically interesting to understand the evolution and speciation of *A. baumannii* when compared to the other species of this genus. Using this analysis, it is possible to infer the origin of specific mechanisms expressed in this species and identify the closest and the most distant members within the genus to standardize comparative analyses better. Phylogenetic analysis based on a few housekeeping genes does not represent the complete evolutionary history or the final diversity between strains or members of the same genus. Nonetheless, a study based on phylogenomics shows a refined ancestry and variety caused by changes in the niche or geographic location of bacterial populations [34].

In comparison, a previous study pointed out that, for a limited number of genes, phylogenetic inference using concatenated genes is better at portraying genetic diversity and distance between different species than the use of consensus trees derived from individual genetic analyses [35]. Therefore, through the proposed method, one can evaluate a significant distancing of *A. baumannii* strains from the other species belonging to this genus, which indicates that the use of concatenated rpoB and 16 S rRNA genes is an excellent option for the inference of phylogenetic distance of strains belonging to specific genera. A previous study obtained a similar result when performing phylogenetic inference using the complete genomes of 136 strains of *Acinetobacter* within the genus [36].

### 3.2. Genomic Plasticity in Acinetobacter Baumannii

Previous work has reported genomic plasticity among persistent *A. baumannii* strains in Italy [14], Argentina [37], and Australia [38]. Historically, it is a species capable of receiving and donating genes to other microorganisms in the environment, a mechanism mediated by recombination events [36].

The analysis of *A. baumannii* genomes revealed the presence of 14 essential elements of resistance within genomic islands that are acquired through the horizontal transfer of genomic recombination events [39]. The largest genomic island found (RI7) has a length of 96,878 nucleotides and presents in its content a total of 25 resistance genes, characterized by an identity of more significant than 70% against the database. This same island is partially shared by strains of different ST 1 and is more similar within strains belonging to ST 2 than ST 1. Previously, this island was described as AbaR1 resistance island, and was considered to be one of the leading genomic elements responsible for the high resistance of the species members due to its size and quantity of elements. Currently, its mobile elements are known to originate from bacteria of the genera *Pseudomonas*, *Salmonella*, and *Escherichia* [1]. Based on several studies, more than 10 islands of resistance have already been identified in the genomes of *A. baumannii* [40]. In the study of the AYE strain, seven islands of resistance were detected.

Similar events of gene displacement and the presence of specific factors related to pathogenicity within genomic islands are also reported in other species with high intrinsic resistance to antimicrobials. The presence of mobile elements containing virulence or resistance factors allows for better adaptation and proliferation of *A. baumannii*. These are usually included in some phylogenetic groups, which have greater global distribution [29]. Therefore, monophyletic clades have stability in gene content, which may explain its low clonal incidence compared to other clones, which are characterized by high genomic plasticity [14]. In the literature, such mechanisms are revealed of transfer and translocation in species such as *Pseudomonas aeruginosa* [41] and *Klebsiella pneumoniae* [42], microorganisms that, like *A. baumannii*, are also considered models for understanding resistomes, virulence, and pathogenicity. This fact indicates that resistance islands are persistent in the distribution of nosocomial bacteria due to selective pressure, and they are spread and fixed in bacteria to generate adaptive fitness.

### 3.3. Functional Characterization through Pan-Genome Analysis

Currently, pan-genome analyses, which allow for the observation of the total genetic repertoire of a species, are incredibly relevant for determining similarity, functional characterization, and analysis of exclusive characteristics of certain strains of a microbial species. Such a report aims to assess the number of genes shared by all representatives of a taxonomic set and the genes shared by more than one, but not all, strains belonging to the group, known as accessory genomes [28]. Presently, pan-genome analyses are relevant for determining genetic variability, similarity, essential genes, functional characterization, and prediction of exclusive genes by phenotypic groups to characterize species and strains, in addition to being able to also view the discrepancies between genomes that are not perceived by conventional analyses [43]. Nowadays, there are reports of pan-genome analyses of several pathogens, such as *Streptococcus agalactiae* [44], *Legionella pneumophila* [45], *Corynebacterium pseudotuberculosis* [46], *Pasteurella multocida* [47], *Pseudomonas aeruginosa* [48], and *Treponema pallidum* [49].

In the pan-genome analysis of *A. baumannii,* a core genome containing 1999 genes was identified. Biologically, using the Kyoto Encyclopedia of Genes and Genomes database, the core genome contains all the essential genes for the survival of the bacteria in a favorable environment. Therefore, it includes pathways related to metabolism and cell division, genetic processes, and energy production [28,49]. Among the genes related to the core genome, only nine are related to resistance, and these genes represent the core resistome.

On the other hand, the accessory genome has genes related to microbial adaptation mechanisms, such as antimicrobial resistance factors, symbiosis, adaptation to the environment, and virulence, which may or may not be acquired via horizontal gene transfer [50,51]. In our study, the accessory genome revealed a total of 10,337 genes that may be related to adaptation to the host and are more represented in pathways of carbohydrate and amino acid metabolism, xenobiotic metabolism, and drug resistance. Considering the pathogenic cycle of the species, xenobiotic biosynthesis and degradation pathways are essential facilitators of bacterial adaptation, mostly when related to microbial antibiosis associated with adaptation to the host [52,53], which, in theory, provides a more prolonged microbial survival. The prevalence of carbohydrate, amino acid, and xenobiotic metabolism pathways comes in part from the pathogen’s evolutionary history [54,55].

In a previous study, Hassan et al. (2016) considered 30 complete genomes of *A. baumannii* for the inference of the pan-genome, reaching values of pan- and core genomes of 7606 and 2445 genes, respectively [56]. Our work suggests a more closed pan-genome (12,336), due to an increase in the core genome (1999). This comparative result was expected, considering the increase in genomes in the dataset. This fact does not invalidate the analysis made previously by the authors but it sheds light on the development of the species’ pan-genome. In contrast, Mangas et al. (2019) considered 2467 complete and draft genomes of the species for inference of the pan-genome, reaching values of the core genome and pan-genome equivalent to 2221 and 19,272 respectively [57]. The result presented in this work tends to present a more closed value in relation to the core genome, which was expected since the exclusive use of complete genomes tends to increase the accuracy of orthologs’ analyses. In contrast, the number of genes found in the pan-genome was lower than that presented by the authors. This fact can be justified by the difference in methodological approaches existing between both works.

### 3.4. Resistome of Acinetobacter Baumannii

In the pan-resistome analysis, it was possible to ascertain the numerical presence of a variant of beta-lactamases, such as the OXA genes. Previous studies have already pointed out that this gene is widespread among the distinct geographic locations of *A. baumannii* strains, reporting that the coding gene for OXA-143 is exclusive for Brazilian strains [58]. Moreover, the same study points out that the enzyme OXA-58 is very prevalent across the globe but has a higher incidence in strains from southeastern Europe [58]. Nevertheless, using the methodology employed, the OXA-143 gene was not detected, but a similar pattern was observed for the beta-lactamase SAT-1, which is exclusive to the Brazilian strain MRSN15313. As for the OXA-58 gene, its presence was inferred for isolated strains in Italy, India, Greece, Ghana, China, and two Mexican strains, indicating and corroborating its higher prevalence in the East.

As for the efflux pumps presented, one of the most important and studied is the *ade*ABC pump, which belongs to the RND family (resistance–nodulation–division) [9]. The same *ade*IJK pump family is adequately represented in the core resistome. Previous studies indicate that efflux pumps are excellent targets for drugs, considering that their inhibition greatly amplifies the action of antimicrobials that, under normal conditions, would be eliminated by the cell [7]. Recent studies report that inhibition of the *ade*B and *ade*J portions leads to a significant reduction in microbial resistance [59]. Both are present inside the cell and anchor the pump to the membrane. As for proteins with enzymatic action, the one that stands out the most is *ampC* beta-lactamase. It has been described with high prevalence in *A. baumannii*, which is considered one of the main species responsible for the resistance to beta-lactams [31].

Interestingly, the preferred treatment for susceptible strains of *A. baumannii* is based on carbapenems [60]; however, in evaluating the pan-resistome, many mechanisms of resistance to this class have been reported, which may suggest that its presence in the genome does not indicate expression, mainly when relating to the presence of resistance mechanisms in the genome of the SDF strain. In the case of resistant strains, tigecycline treatment has been used with varying success [10,60].

Still, in this context, it is known that the number of reports of strains resistant to polymyxins within the species has grown [61,62]. However, it was possible to predict only one gene related to the resistance phenotype to that class of antimicrobials based only on the predicted proteome of the species.

## 4. Materials and Methods

### 4.1. Genomes Database, Annotation, and Data Retrieval

All the complete *A. baumannii* genomes and their plasmids were obtained through the National Center for Biotechnology Information (NCBI)/GenBank-RefSeq [63]. An in-house Python3 script was developed to extract chromosome sequences from strains with plasmid sequences, using as a criterion the extraction of the largest contig present in the fasta file. Both files, those containing only the chromosome and those containing the chromosome and plasmid, were annotated using the same parameters in the PROKKA pipeline version 1.13.7 [64], with an additional setting: the prediction of RNAs, using RNAmmer software version 1.2 [65].

### 4.2. Multilocus Sequence Typing and Phylogeny

The similarity analysis was performed using all the complete genomes as input to the software FastANI [21], using default parameters. The sequence type was predicted using the MLST 2.18.0 software, based on the PubMLST platform [66]. The scheme used was the *abaumannii_2*, determined and made available by the Pasteur Institute based on seven sequenced housekeeping alleles: *cpn60* (Chaperonin family protein), *fusA* (Elongation factor G), *gltA* (Citrate synthase), *pyrG* (CTP synthase), *recA* (Protein RecA), *rplB* (50S ribosomal protein L2), and *rpoB* (a beta subunit of RNA polymerase) [67].

A customized Python3 script was used to extract the nucleotide sequences of 16S rRNA and rpoB genes, ranked by BLAST similarity (>98%) against *A. baumannii* AYE reference sequences. Subsequently, the extracted sequences were concatenated into a single file. The phylogeny was performed using the maximum likelihood method using the *rpoB* and 16S rRNA sequences. The alignment was performed using MAFFT software version 7.31.0 with default parameters [68], and the phylogenetic tree was inferred with the MEGA7 software [69], using the maximum likelihood method with statistical support of 10,000 bootstrap iterations to amplify the reliability of the formed clades. The generated tree figure was optimized using the FigTree 1.4.4 software [70].

### 4.3. Resistance Genes Profile

The Comprehensive Antibiotic Resistance Database (CARD) [11] was used to compare the local alignments and the determination of the presence of genes related to microbial resistance. For this purpose, the predicted proteome product of the automatic annotation of *A. baumannii* (206 strains) was used.

A customized Python 3.6 script was used to automate BLAST alignments [71] of proteomes against the CARD database. Only the results whose identity and coverage were equal to or greater than 70% and an E-value below 5 × 10^−6^, respectively, were used. It was also used to generate the binary matrix of presence and absence genes, considering the previous mining files of the multiple alignments [72]. The final result was to generate the cluster map. The prediction of plasmid resistome was possible by comparing the annotation of the complete genomes (chromosome and plasmid) with the annotations of the chromosome only. The statistical difference related to the number of resistance factors between the strains that do not have a plasmid and those that do have a plasmid was made by the Wilcox-on-Mann–Whitney test.

### 4.4. Genomic Islands Analysis

Genomic Islands Prediction Software (GIPSy) [39] was used to perform the prediction of the genomic islands. In this analysis, the AYE strain was selected for the reference genome due to its history of resistance on the European continent and the high presence of resistance genes. As a subject, the genome of *A. baumannii* SDF was selected, which is a strain previously described as susceptible [1,39,73,74]. Subsequently, the BLAST Ring Image Generator (BRIG) software was used to visualize the genomic islands present in the genomes [75].

### 4.5. Pan-Genome and Pan-Resistome Analyses

The significant pan-genomic analyses were performed using the software Orthofinder [76]. It uses MCL (Markov Clustering algorithm) to determine the clusters of orthologous genes based on multiple alignments using the amino acid fasta as input. For the pan-genomic analysis, the annotation of both chromosomes and plasmids was considered. For the functional analysis of each subpartition, multiple comparisons against the Kyoto Encyclopedia of Genes and Genomes database (KEGG) [77] were considered. Obtaining the values related to the development of the pan-genome, as well as the alpha value was done through an in-house script.

The extracted core genome, resulting from the analysis using Orthofinder, was aligned with MAFFT [68] for subsequent phylogenomic inference using FastTree software with maximum likelihood methodology.

## 5. Conclusions

There is a wide variety of genes in the total repertoire of the species studied. Unfortunately, there is no visible clustering for the host and geographic location; however, the grouping of the strains based on ST reveals a coherent pattern, corresponding to the core genome similarity. The repertoire of the resistome was characterized in terms of the presence and similarity of genes in the total pan-genome. It demonstrated enormous plasticity when evaluating the distribution of factors throughout the groups and the analyzed phylogeny. The pan-resistome also pointed out the presence of the *ade*IJK efflux pump and *amp*C enzyme in all the strains of this species, as well as the heterogeneous distribution of resistance factors across the globe. Another interesting fact is the higher amount of resistance factors to cephalosporins, aminoglycosides, and tetracycline in the studied genomes. Therefore, there is a contraindication to the use of these drugs in *A. baumannii.* These facts point mainly to the discrepancy of strains belonging to different STs within the *A. baumannii* species and its high capacity to remodel the gene repertoire to adapt to the environment or host, and, hence, can remain as an important pathogen for years. Therefore, the data collected are pertinent to better evaluate the high resistance of the species in a hospital environment and, consequently, can be used for a targeted prescription of antibiotics based on phenotyping related to a genetic presence profile. From this perspective, it is possible to use the data obtained in this work to carry out studies for new drug candidates based on the core genome and to take advantage of the assembled pan-resistome to anticipate possible escape mechanisms of *A. baumannii*.

## Figures and Tables

**Table 1:** Description of the genes present in the core resistome of the studied strains containing the mechanisms of action and antibiotics associated with these mechanisms [11].

Gene	Definition	Mechanism	Antibiotic
adeK	The outer membrane factor protein in the adeIJK multidrug efflux complex	Antibiotic efflux	Phenicol, rifamycin, penem, diaminopyrimidine, tetracycline, carbapenem, macrolide, lincosamide, floroquinolone, cephalosporin
adeJ	An RND efflux protein that acts as the inner membrane transporter of the AdeIJK efflux complex	Antibiotic efflux	Diaminopyrimidine, phenicol, tetracycline, rifamycin, carbapenem, penem, fluoroquinolone, macrolide, cephalosporin, lincosamide
adeI	The membrane fusion protein of the AdeIJK multidrug efflux complex	Antibiotic efflux	Phenicol, rifamycin, penem, diaminopyrimidine, tetracycline, carbapenem, macrolide, lincosamide, floroquinolone, cephalosporin
adeF	The membrane fusion protein of the multidrug efflux complex AdeFGH	Antibiotic efflux	Tetracycline, fluoroquinolone
adeG	The inner membrane transporter of the AdeFGH multidrug efflux complex.	Antibiotic efflux	Tetracycline, fluoroquinolone
adeL	A regulator of AdeFGH in *Acinetobacter baumannii*. AdeL mutations are associated with AdeFGH overexpression and multidrug resistance.	Antibiotic efflux	Tetracycline, fluoroquinolone
ampC	AmpC type beta-lactamases are commonly isolated from extended-spectrum cephalosporin-resistant Gram-negative bacteria.	Antibiotic inactivation	Cephalosporins
adeN	AdeN is a repressor of AdeIJK, an RND-type efflux pump in *Acinetobacter baumannii*. Its inactivation increases the expression of AdeJ.	Antibiotic efflux	Carbapenem, diaminopyrimidine, rifamycin, penem, tetracycline antibiotic, phenicol, lincosamide, fluoroquinolone, cephalosporin, macrolide
abeM	AbeM is a multidrug efflux pump found in *Acinetobacter baumannii*.	Antibiotic efflux	Acridine dye, fluoroquinolone antibiotic, triclosan

## Data Availability

The dataset used in this study is available in Supplementary Table S1.

## Supplementary Materials

The following are available online at https://www.mdpi.com/article/10.3390/antibiotics10050596/s1, Figure S1: Cluster map representing the genomic similarity evaluated among all strains of *Acinetobacter baumannii* included in the study. The intensity of the color indicates a greater degree of similarity between the genomes. For each strain, the isolation site and the predicted sequence type were added respectively. The cladograms were generated using Euclidean distance. Figure S2: Phylogenetic tree based on the concatenated sequences of the 16S rRNA and rpoB genes representing the positioning of the *Acinetobacter baumannii* strains compared to the genus. Statistical support of 10,000 bootstraps was applied. The colors represent different species within the genus *Acinetobacter*. Figure S3: Cluster map representing the presence of resistance genes (*X*-axis) in all genomes (*Y*-axis) were addressed. In the case of existence, the color intensity represents the sequence similarity to the database used, with a minimum similarity of 70% versus the CARD database. The cladograms used are based on the Euclidean distance between the data. Table S1: Genomic data on the deposit made available by the National Center for Biotechnology Information (NCBI). The information is distributed respectively in Strain, BioSample, BioProject, Assembly, Size, GC%, and FTP for RefSeq access. Table S2: Matrix representing the pan-resistome of the strains under study. Numbers >0 represent the presence and similarity to the CARD database of the gene in the genome of the strain presented in the first column. The strains were grouped according to the phylogenomic proximity of the core genome. The first column represents the local isolation of each strain. The second column represents the sequence type predicted. Table S3: Resistance genes predicted exclusively in plasmids. The relative presence of genes considers only the 162 strains deposited with plasmid.
