# Supplementary material for: Pan-Resistome Insights into the Multidrug Resistance of Acinetobacter baumannii
Source: Antibiotics (Basel). 2021 May 18;10(5):596. doi: 10.3390/antibiotics10050596 (PMC8157372; doi:10.3390/antibiotics10050596)
Supplement: Supplementary file 1 [file antibiotics-10-00596-s001.zip › Supplementary Figure S2.pdf]

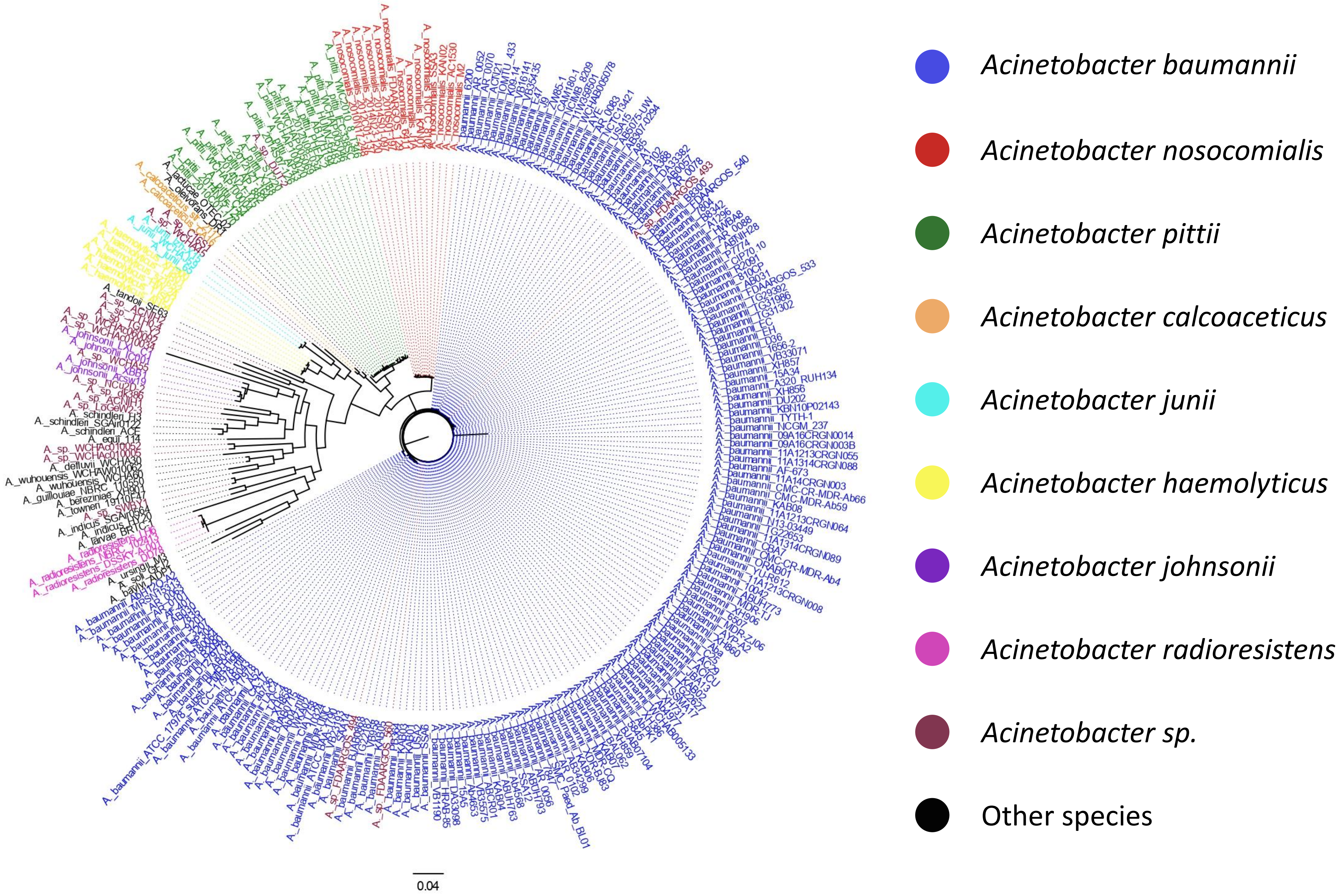
0.04
Acinetobacter baumannii
Acinetobacter nosocomialis
Acinetobacter pittii
Acinetobacter calcoaceticus
Acinetobacter junii
Acinetobacter haemolyticus
Acinetobacter johnsonii
Acinetobacter radioresistens
Acinetobacter sp.
Other species
