# Supplementary material for: Pan-Resistome Insights into the Multidrug Resistance of Acinetobacter baumannii
Source: Antibiotics (Basel). 2021 May 18;10(5):596. doi: 10.3390/antibiotics10050596 (PMC8157372; doi:10.3390/antibiotics10050596)

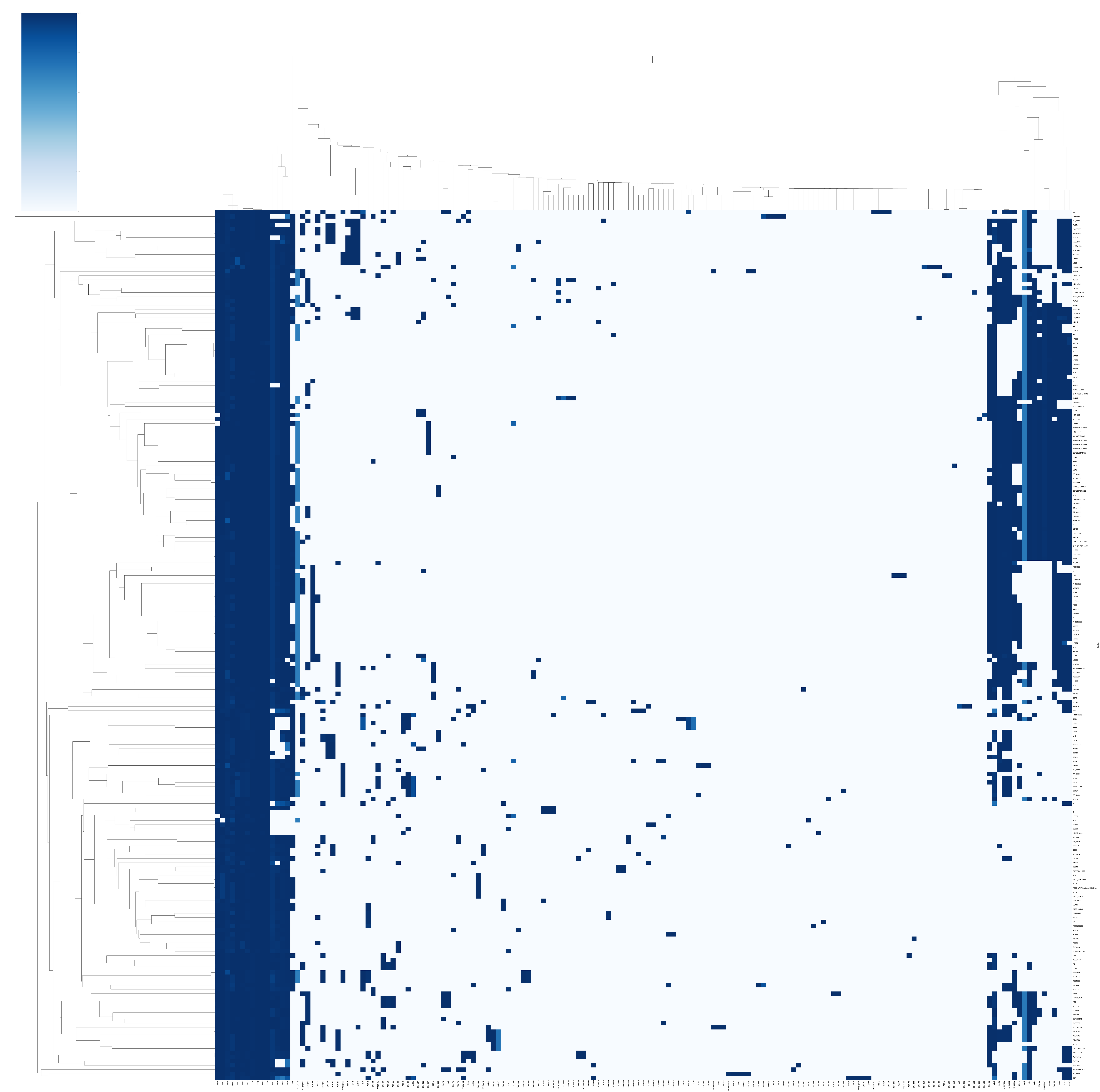

Supplement: Supplementary file 1 [file Supplementary Figure S3.pdf › Supplementary Figure S3.pdf]
